# What was the population of Great Zimbabwe (CE1000 – 1800)?

**DOI:** 10.1371/journal.pone.0178335

**Published:** 2017-06-14

**Authors:** Shadreck Chirikure, Thomas Moultrie, Foreman Bandama, Collett Dandara, Munyaradzi Manyanga

**Affiliations:** 1 Department of Archaeology, University of Cape Town, Cape Town, South Africa; 2 School of Management Studies and Centre for Actuarial Research, University of Cape Town, Cape Town, South Africa; 3 Division of Human Genetics, University of Cape Town, Cape Town, South Africa; 4 Department of History, University of Zimbabwe, Harare, Zimbabwe; Seoul National University College of Medicine, REPUBLIC OF KOREA

## Abstract

The World Heritage Site of Great Zimbabwe is one of the most iconic and largest archaeological settlements in Africa. It was the hub of direct and indirect trade which internally connected various areas of southern Africa, and externally linked them with East Africa and the Near and Far East. Archaeologists believe that at its peak, Great Zimbabwe had a fully urban population of 20,000 people concentrated in approximately 2.9 square kilometres (40 percent of 720 ha). This translates to a population density of 6,897, which is comparable with that of some of the most populous regions of the world in the 21^st^ century. Here, we combine archaeological, ethnographic and historical evidence with ecological and statistical modelling to demonstrate that the total population estimate for the site’s nearly 800-year occupational duration (CE1000–1800), after factoring in generational succession, is unlikely to have exceeded 10,000 people. This conclusion is strongly firmed up by the absence of megamiddens at the site, the chronological differences between several key areas of the settlement traditionally assumed to be coeval, and the historically documented low populations recorded for the sub-continent between CE1600 and 1950.

## Introduction

**Fig 1:**
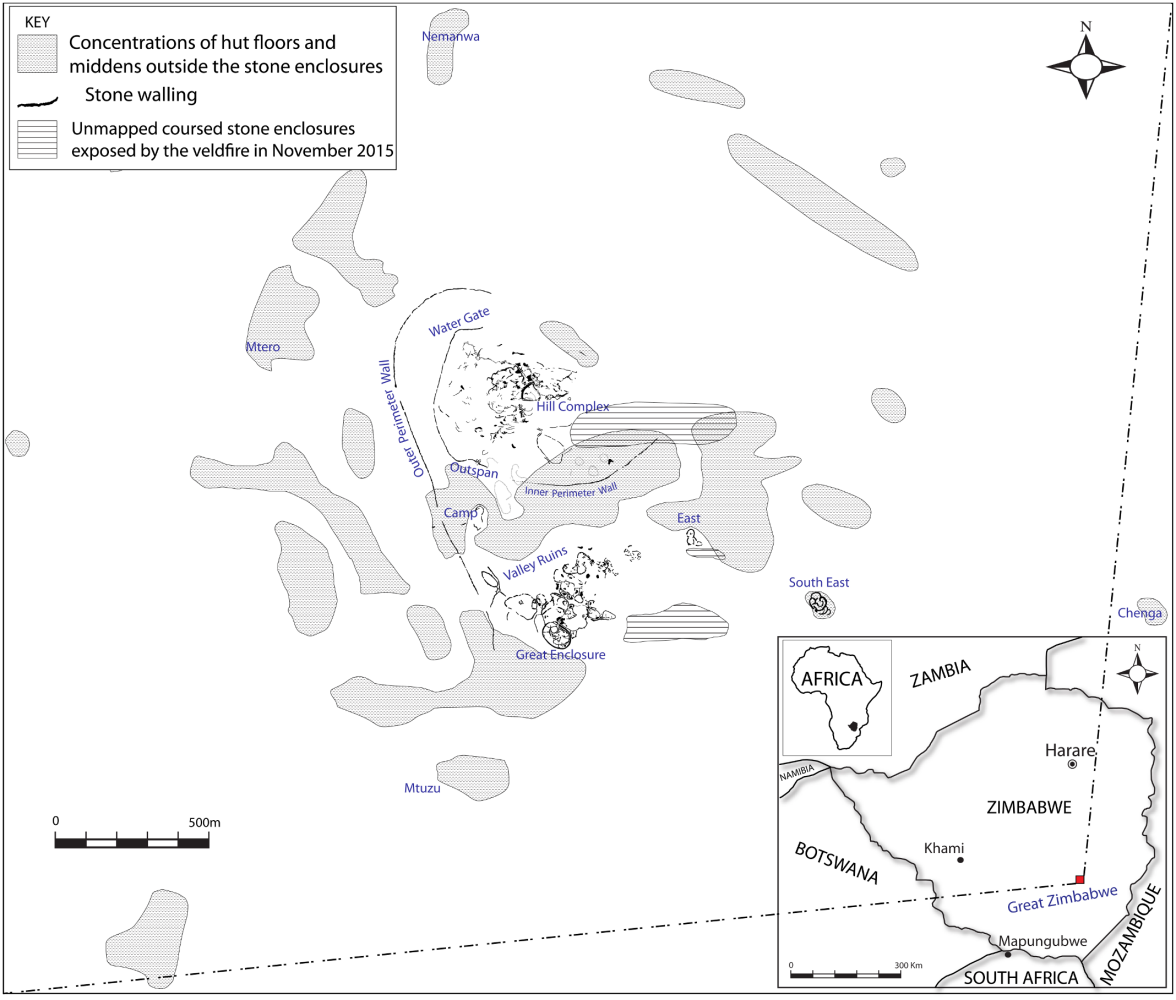
The location of Great Zimbabwe.

Achieving sustainability in the short- and medium- to long term has emerged as a major global aspiration for the planet. Necessarily, the demography of prehistoric and contemporary populations occupies an important role in inter-disciplinary and multi-scalar discourses on sustainability. Fast-growing populations, when considered in light of scarce resources, are a major challenge for regions such as Africa. Because it was experienced, the past is often a platform on which long-term planning rests. Therefore, the population of major precolonial African centres with extensive local and international networks such as Great Zimbabwe (Fig 1), when credibly estimated, ought to play a significant role in understanding past and future societies.

**Fig 2:**
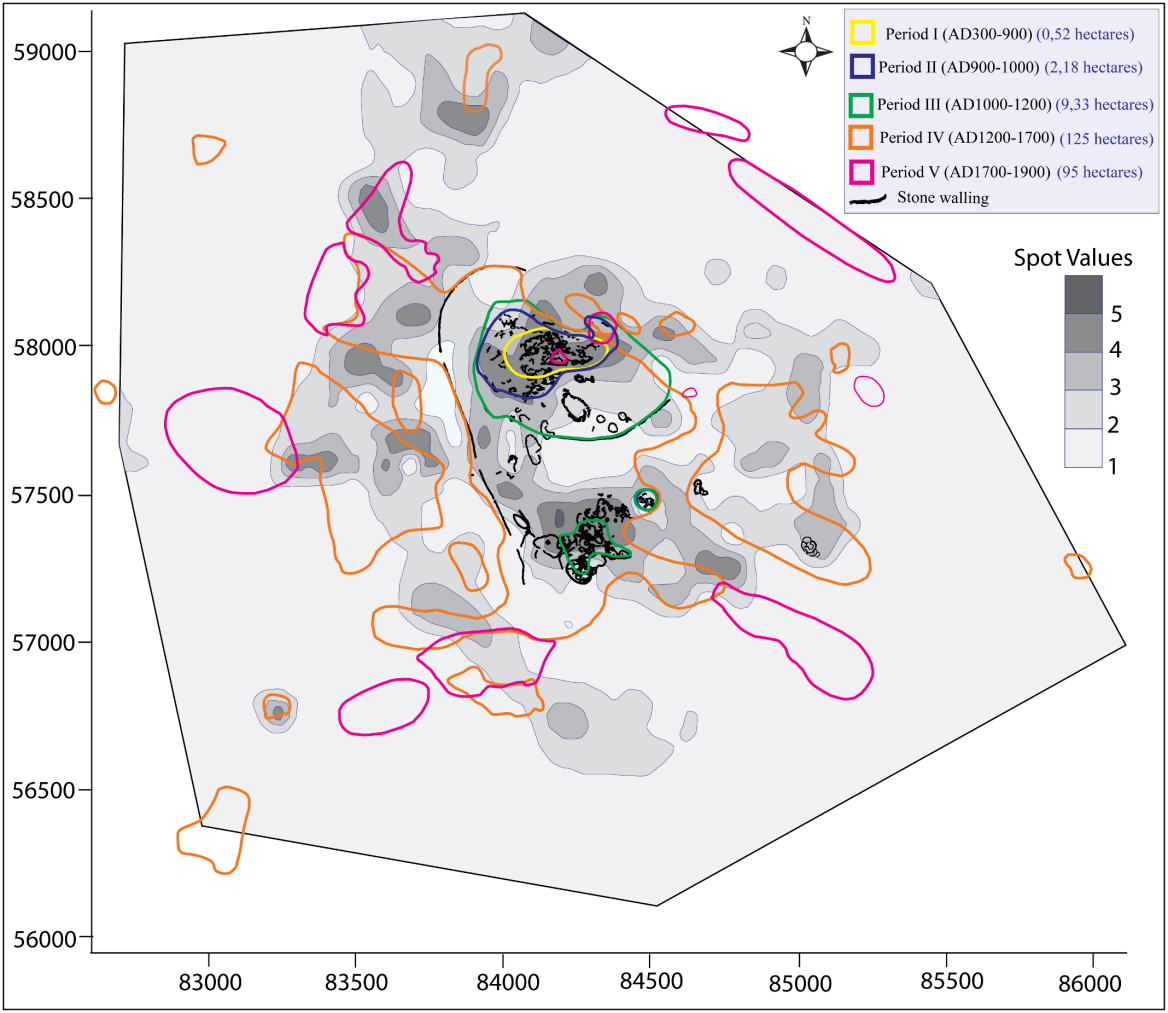
Distribution of the major periods of occupation at Great Zimbabwe overlaid with a phosphate map of the site (adapted from [2, 6]).

In contemporary southern Africa, such an aspiration is, however, stillborn, attested by the absence of high quality prehistoric demographic data including estimates. Here, the default position is that Great Zimbabwe was occupied by 20,000 people during its peak, previously believed to be between CE1300 and 1450 [1]. Combined data from phosphate and geophysical magnetic surveys indicates that only 40 percent of the 720 ha making up Great Zimbabwe has evidence of permanent settlement remains (Fig 2) [2, 3], converting into an implied population density of 6,897 people per square kilometre. In 2016, the population density of Hong Kong was 6,996 per square kilometre, while the 2013 population density for New York City was 10,292 people per square kilometre [4]. Khayelitsha, a populous low-income township in Cape Town had a 2016 population density of 7,748 people per square kilometre, with an average household size of four [5]. In the same year, the modern town of Masvingo only 28 kilometres north-west of Great Zimbabwe had a population density of 1,900 per square kilometre [5]. Even if the entire 720 ha of Great Zimbabwe site was occupied in its entirety, a population density of 2,778 per square kilometre still compares favourably with that of a high number of modern towns scattered across Africa, and in many of the world’s regions [5]. Whether Great Zimbabwe had such a high population density or not is one thing, but the ecological and sustainability implications of continuous occupation by 20,000 people in a fairly restricted area are yet to be fully considered.

The very first population censuses conducted by the colonial administrators in the late 19^th^ and early 20^th^ centuries add more weight to these observations. In 1895, the population of Southern Rhodesia (Zimbabwe) was estimated to be just under 600,000 and, by 1922, had increased to 880,000 [7]. Manning et al. [8] estimate that the population of the vast region between the margins of the Kalahari Desert in eastern Botswana and central Mozambique was 1,9 million in CE1600. Furthermore, the same database suggests that the combined population of the massive region made up of modern day Zimbabwe, Namibia, Botswana, South Africa, Mozambique, Zambia and Malawi was less than that of Nigeria at the time, and is still so [9]. Without interrogating the archaeology on the ground, these basic consistency checks expose, rather strongly, the necessity of scientifically grounded estimates for Great Zimbabwe’s population. Here, we develop a reproducible demographic estimate for Great Zimbabwe informed by archaeological, historical, and ethnographic observations. When combined with demographic back projection and ecological modelling, the picture that emerges is that of a very low population that is consistent with historical demographic data sets of the period CE1600 to 1960.

### Great Zimbabwe: A brief background

Great Zimbabwe is located on the southern fringes of southern Zambezia: the region bounded to the east by the Indian Ocean, to the west by the Kalahari Desert, to the north by the Zambezi River, and to the south by the Soutpansberg range of mountains. One of the most significant cultural innovations that developed in this region between CE1000 and 1900 is the Zimbabwe culture whose prominence is attested by the presence of more than 1,000 residences built of drystone walls without any binding mortar [10, 11, 12]. Great Zimbabwe, at one time the capital of a powerful state which ruled a sizeable territory in this sub-region, is arguably the most globally famous Zimbabwe culture site [13]. Its size, monumentality, and evidence of internal and external trade, all contributed to its attraction amongst researchers [14, 15]. The international significance of the Zimbabwe culture is brought into sharp relief by the fact that three of its former capitals, Khami, Mapungubwe and Great Zimbabwe, are inscribed on the UNESCO World Heritage List.

**Fig 3:**
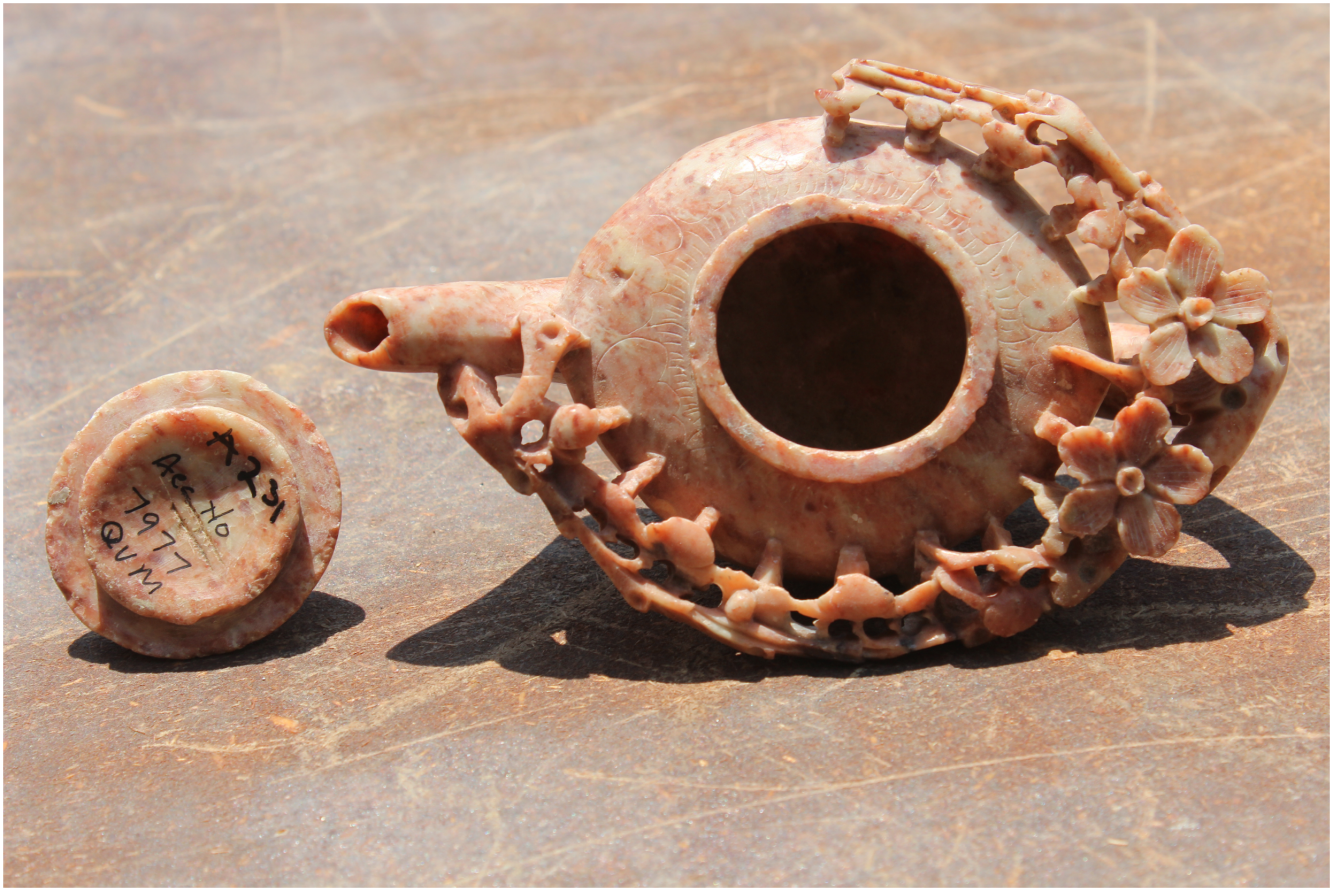
Chinese jade teapot found from the renders ruin at Great Zimbabwe.

**Fig 4:**
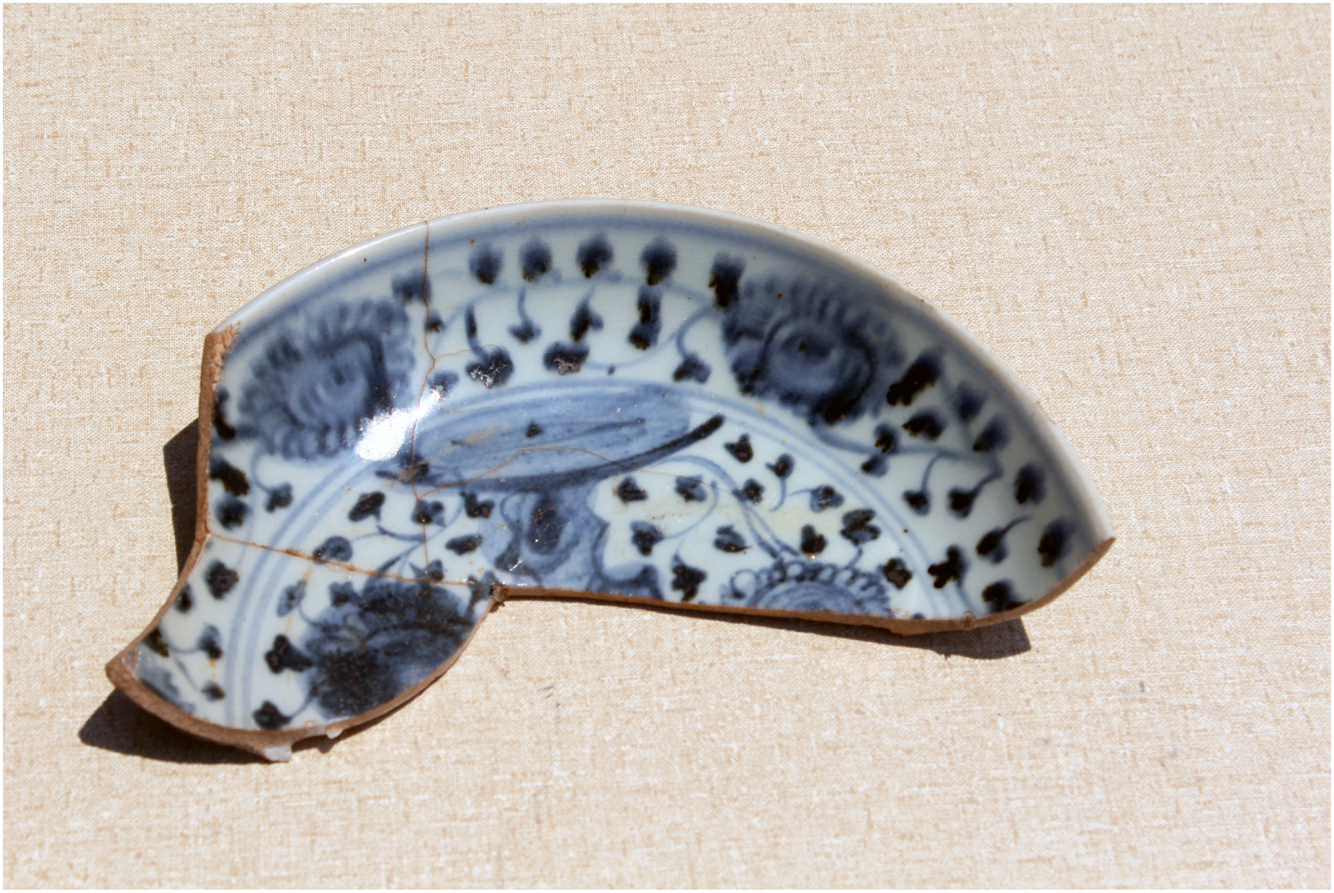
Chinese blue on white porcelain found in the Lower Valley enclosures at Great Zimbabwe.

Great Zimbabwe is comprised of walled areas such as the Hill Complex, the Valley and Great Enclosures, nearby smaller walled areas, and surrounding unwalled settlements, spread over 720 hectares (Fig 1) [2]). The evolution of the occupation associated with the builders of stone walls at Great Zimbabwe passed through four different phases: Periods II (CE900–1000), III (CE1000–1200), IV (CE1200–1700), and V (CE1700–1890) [6, 16] (Fig 2). This division is only useful for analytical purposes because in practice it is difficult to separate the end of one phase from the beginning of a new one. From the early second millennium CE onwards, exotic objects such as glass beads, Chinese celadon, Chinese porcelain and Islamic glass and fritware, when coupled with iron gongs from central Africa, demonstrate that Great Zimbabwe participated in internal and external long-distance trading networks (Figs 3 and 4) [14].

After a 20^th^ century dominated by the need to confirm, at least in the Western mind, that Great Zimbabwe was local in origin, Garlake ([14] p.195) was the first researcher to estimate the population of Great Zimbabwe. By that time, it was generally believed that the upper classes at the site occupied areas with stone walling while the commoners were consigned to unwalled areas [10]. To arrive at what might be regarded as the first scientific estimate of Great Zimbabwe’s population, Garlake calculated the average number of houses in a typical walled settlement, and moderated the calculation by comparison with evidence from similar sites elsewhere. The distribution of settlement remains suggested to Garlake that the unwalled area at Great Zimbabwe was three times bigger than that of walled sections. Influenced by the ethnographic thinking that between one-and-a-half to two huts belonged to an adult, Garlake suggested that the total population of Great Zimbabwe was somewhere between 1,000 and 2,500 people, a tenth of whom resided within the walls [14].

On excavating an unwalled settlement near the Camp Ruins, Huffman [17] estimated the population of Great Zimbabwe by extrapolating the number of huts found inside and outside the stone walls across the site. Using a ratio of one adult to one hut, and a comparison with a late 1960s population pyramid for Rhodesia (now Zimbabwe), an estimate of 11,000 people during Great Zimbabwe’s peak was computed. In the late 1980s, Huffman ([18] p. 323) arrived at a minimum population of 18,000 during the site’s peak by applying early colonial demographic figures for eastern Zimbabwe during the early colonial period (late 19^th^ and early 20^th^ centuries), to the procedure above. While the procedure adopted by Huffman is not readily reproducible, there is one major particular point at which his assumptions and overall population estimate diverge. A critical analysis of the 1960s population pyramid used to estimate the precolonial population indicates that the demographic base on which to extrapolate the total population of the site is likely to be significantly overstated. In 17^th^ century Zimbabwe, life expectancy at birth would have been of the order of 25 to 30 years, characterising a population experiencing both high fertility and exceptionally high levels of child mortality (where approximately 40 percent of children would not survive to their fifth birthday). Stable population theory (allowing for population growth of 10 per mille) suggests that in a population with that level of child mortality, the number of children under the age of 15 years would be around 60 percent of the population of adults over that age. The population pyramid of then-Rhodesia in the 1960s would have been markedly different, and suggest a much higher proportion of children relative to adults. Noteworthy is the fact that Huffman’s source, ([19] p. 66), suggests almost equal numbers of children and adults. This, too, would have had the effect of overestimating the population when applied to Great Zimbabwe. Regardless of these serious oversights, the high estimate of 18 000 people started to seep unquestioned into both the historical and archaeological imagination, and into school textbooks and public knowledge [20]. Presently, in the 21^st^ century, the population estimate for Great Zimbabwe is now unquestioningly accepted as being between 18,000 and 20,000 people.

While generally accepted knowledge reflects the state of research at any given point, old and new chronological, archaeological, and historical evidence, however, indicates that not all the components of Great Zimbabwe were built and occupied at the same time (Fig 2) [21, 22]. For example, settlement in the modern Car Park (CE1450–1660), previously assumed to be a commoner residence, post-dates the main occupation on the Hill Complex [22]. Likewise, the Lower Valley enclosures were occupied only when the Great Enclosure and other Upper Valley enclosures were abandoned [21]. Because the generally accepted population of 20,000 does not factor in chronological differences between various areas, it becomes clear that any useful scientific estimate of Great Zimbabwe’s demography must be anchored in good temporal controls that consider overlaps and disjunctures in occupation to avoid under- or overestimating the population of the site. Given the fragmentary nature of archaeological evidence, and that to date no burials have been recovered from the site, multiple consistency checks are essential as part of any model development and interpretation.

## Data sources and methodology

The study did not involve any invasive techniques. Permission to conduct research was granted by the National Museums and Monuments of Zimbabwe.

### a. Ethnography

Great Zimbabwe is a former Shona settlement that is interpreted using ethnohistorical and ethnographic observations. Shona households typically live in a homestead with clearly-defined built living spaces: kitchen, bedroom, dormitory for girls, dormitory for boys, and visitors’ house [23]. Although the number of houses per homestead varied depending on whether a family was monogamous or polygamous, these key spaces were always present. In a polygamous family, each wife often had her own kitchen, bedroom and granaries [23]. Our own fieldwork in rural Gwanda, south-western Zimbabwe revealed that the average homestead size is 0.36 hectares in spatial extent. The average floor area of living huts was 12.57 square metres of living space. However, an individual needs 10 square metres of roofed space (according to the Naroll’s constant) [24]. Generally, the average household size in 21^st^ century rural Zimbabwe is four people [25]. Elsewhere in the world, household sizes of agricultural communities tend to fall within the range three to seven individuals with a median value of five [24]. Using this ethnographic information, [23, 24, 25], the number of homesteads in walled and unwalled areas were estimated (see below). The first assumption (Model 1) was that each hut at Great Zimbabwe was occupied by a minimum of four and a maximum of seven people. Model 2 assumed that a homestead of five structures housed only a single household of four (local average) and seven people (international average).

### b. Archaeology

No burials were recovered from Great Zimbabwe. Our population estimates were anchored in settlement and house remains. Initially, the number of houses known in walled and unwalled areas (Fig 1) was estimated by occupation period. It must be noted that the assumption that Great Zimbabwe was a rainmaking centre in Periods I and II [1] has been challenged [15] because the material culture ascribed to rainmaking, such as the Period II crucibles which appear in stratified contexts is more constistent with normal homesteads than shrines [16, 15]. Of all the walled areas, the Maund Ruins area was totally excavated and exposed 10 huts per area, that is, approximately 0.25 ha [26]. In enclosures where no hut numbers are in the literature, polygons were created in Google Earth to calculate the total area. The total number of huts was estimated using the ratio of 10 houses per 0.25 ha.

The estimate of the number of huts in the unwalled areas factored in the assumption that houses in unwalled areas were closely packed. In the area around the modern Curio Shop, 30 hut floors were exposed, which is about three times the number of huts identified in any enclosure [1]. However, a critical examination of some of the floors demonstrated that they are too small to have been living spaces. Rather, some walls formed arcs possibly to demarcate courtyards, mimicking the layout in the walled areas. The only difference was that earthen structures or *dhaka* replaced stones. This makes an estimate of 30 houses too high. However, to allow for permutation of error, the high number of 30 huts per 0.25 hectares was retained.

**Fig 5:**
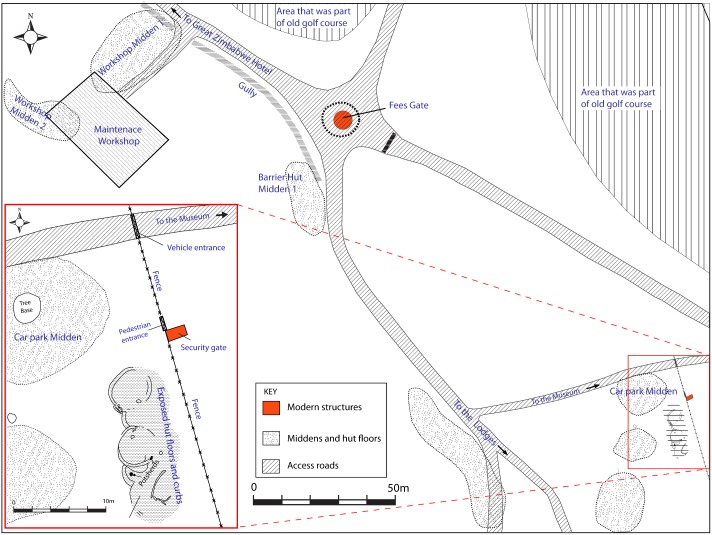
A section of the western side of Great Zimbabwe including the Car Park and Maintenance Workshop areas. The areas where hut concentrations were observed, is also shown.

Non-invasive field walking and recording by tape measure and GPS was carried out in different areas with no stone walls. The main observation was that there were densities of hut clusters, varying in number between three and five, located with various distances ranging from two to 50 metres apart. The only limitation of this ground truthing was that observations were limited to what is archaeologically observable (Fig 5). However, it was clear that the concentrated nature of huts around the Camp Ruins (Curio Shop area) was probably an exception and not the norm. Furthermore, the stratigraphy of the Curio Shop houses has never been published such that it is not clear whether all the excavated houses dated to the same or different periods. Because Great Zimbabwe’s unwalled areas experienced different activities in the 20^th^ and 21^st^ centuries, a decision was made, after obtaining permission, to clean up exposed sections in different areas to estimate the depth of deposit. The Car Park Midden area had a maximum depth of 80 cm, the same as the Maintenance Workshop area, while the Fire Guard at the back of the Great Enclosure had a maximum depth of 60 cm, the same as the area around the Mujejeje at the back of the Eastern Enclosures. Therefore, the depth of deposit in the unwalled areas was not as great as that in the walled areas, which was between two and four metres [16].

In all cases, the main observation was that the layout of the houses found on the unwalled areas was like that of the walled areas but solid earthen walls substituted for stone walls. To produce a systematic estimate per unwalled area, polygons were drawn in Google Earth to measure the spatial extent of hut floors and material culture concentrations. To allow for permutation of error from both the field walking and recording, the ratio of 30 huts per quarter of a hectare was used to estimate possible numbers of houses in unwalled areas (Table 1). Site extent was determined using a combination of phosphate mapping and geophysics survey [2], and detailed field walking [6]. Fig 2 shows that within the 720 ha estate making up present-day Great Zimbabwe, approximately 40 percent has settlement evidence in the form of floors or middens [2, 6]. Sinclair and colleagues cored various areas of Great Zimbabwe and outlined the approximate spatial extent of various occupation periods [2, 3]. When added to observations from our own fieldwork, it became possible to estimate Great Zimbabwe’s population by period. The archaeological information was combined with ethnographic insights to develop the two demographic estimation models presented below.

**Table 1:** Estimated number of houses for the walled and unwalled areas based on surface indications, data from published excavations and estimates of general extent of individual occupation periods.

Period	Total area (ha)	Est. no. of huts	Sources
Period I	0.52	20	Own estimate
Period II	2.18	87	Own estimate
Period III	9.33	137	Own estimate
Period IV	125	287	Own estimate [14, 16, 21]
Period V	95	158	Own estimate [14, 16, 21]
Total	232	689	

### c. Historical population censuses and estimates

The approach adopted here was to make use of the data provided by Frankema and Jerven [9] for Zimbabwe’s population from 1850–1960. Tabulations of the population by district in 1921 presented in Beach [27], however, suggesting an undercount of around 50 percent relative to Frankema and Jerven’s estimates for 1920, indicate that the population of Victoria District (in which Great Zimbabwe is located) comprised around 4.6 percent of the national population (c. 800,000). To err on the side of conservatism, we have assumed that Victoria’s population was 5 percent of the national, and that—following Beach—the spatial distribution of the population was largely unchanged in the precolonial era. To estimate the population size in 1600, we assumed (on the high side) that population growth, which was just over 10 per mille in 1850, fell linearly over each decade to two per mille in 1770, remaining constant at that level before that date. This suggests a total population of around 275,000 in 1600. To set a lower estimate, we assume that population growth fell linearly over each decade to one per mille in 1680 and was at that level prior to this date. This produces a population of around 230,000. Applying the factor of 5 percent to estimate the population of Victoria District produces a population range of 11,500 to 13,750 in 1600.

## Results

### Estimation models

Our two estimation models (Model 1 and Model 2) and associated consistency checks indicated that the population of Great Zimbabwe remained low, with a steady increase throughout the second millennium CE. Assuming that every hut at the site was occupied at the same time by four (local household average [25]) and seven (international household average [24]) people, Model 1 suggests (assuming that the all areas of the site were occupied at the same time) that the population of the entire site would plausibly have been between 2,756 and 4,823 (Table 2). Deconstructing these global figures by occupation phase is essential to factor in chronological realities within a stable population framework. Owing to its localised nature, it is likely that there were 20 Period I huts, translating to 80 (based on four people per hut) and 140 (seven people per hut) inhabitants. The 87 Period II houses confined to the hill and its terraces implies a population of 348 and 609. Period III saw the expansion of settlements into the Valley and Great Enclosure areas. Adding the total number of huts (137) in all Period III converts to a minimum population of 548, and a maximum of 959 people.

**Table 2:** Estimated number of huts for different components of Great Zimbabwe and corresponding population estimates based on the extreme assumption that every hut had a minimum of four and a maximum of seven people (Model 1) (see Fig 2 for the distribution of occupation periods).

Period	Total area (ha)	Est. no. of huts	Total no. people @4 per hut	Total no. of people @7 per hut
Period I	0.52	20	80	140
Period II	2.18	87	348	609
Period III	9.33	137	548	959
Period IV	125	287	1,148	2,009
Period V	95	158	632	1,106
Total	232	689	2,756	4,823

In Period IV, the estimated 287 houses translate into 1,148 and 2,009 people. Settlements initially excluded unwalled areas outside the perimeter wall and the areas which are exclusively built with Q walling, such as the Maund Ruins. Subtracting the houses in these areas, the population would initially have been 588 (min) and 1,029 (max). When the Great Enclosure, Hill Complex and Western Valley enclosures were abandoned the population would have been 640 (min) and 1,120 (max). The 158 Period V huts convert into a minimum of 632 and a maximum of 1,106 people. However, this model assumes that four or seven people shared 12.57 square metres (average area of a single hut at Great Zimbabwe) when the average amount of roofed space per person elsewhere is, according to the Naroll’s constant, 10 square metres [24]. It is almost impossible to tessellate more than four adults occupying 1.8 m x 0.8 m each, and four smaller children, occupying 1 m x 0.6 m each, into a hut that is 12.57 square metres in size, which renders the implied figures improbable.

Model 2 (Table 3) factored in local realities and initially estimated the total number of homesteads per area based on the ethnographic indication that individual Shona homesteads and individual households had on average five structures used for living space. The population was estimated based on a minimum household of four and maximum of seven people per homestead. Assuming that Great Zimbabwe was occupied at the same time, this model shows that the population would have been 548 (min) and 959 (max). Based on an estimate of 20 huts, there would have been 16 and 289 people during Period I. Because of its concentration on the hill and its terraces, the assumption is that there were 17 Period II homesteads, yielding respectively minimum and maximum populations of 68 and 119. This population would have increased to 108 and 189 in Period III. For Period IV, the population would have been respectively 228 and 399, assuming that the entire site was occupied. When the Hill, Western Valley and Great Enclosure were the only places occupied, the population would have been 100 and 175. When these places were abandoned and new areas were added, the population would have been 128 and 224. However, this requires several consistency checks for moderation.

**Table 3:** Population estimates for Great Zimbabwe based on the number of homesteads and averages of four and seven people per household (Model 2) (see Fig 2 for the distribution of occupation periods).

Period	Total area (ha)	Est. no. of huts	Est. no. of homesteads	Total no. people @4 per hut	Total no. of people @7 per hut
Period I	0.52	20	4	16	28
Period II	2.18	87	17	68	119
Period III	9.33	137	27	108	189
Period IV	125	287	57	228	399
Period V	95	158	32	128	224
Total	232	689	139	548	959

### Consistency checks

Other evidence can be marshalled to suggest that the estimate of 20,000 people in Great Zimbabwe is too high. Five different approaches can be used: demographic back-projection of the population of the region from the early 20^th^ century; assessment of land available for agriculture; the land required to sustain cattle consistent with pouplation of that size; evidence of iron smelting; and the quantity of material culture objects unearthed at the site.

### Demographic projection

Precolonial demographic data of any form in Zimbabwe are non-existent, while data from the colonial period are highly problematic—for reasons of both completeness and accuracy [27, 28]. The quantitative evidence that can be marshalled is therefore, at best, tenuous. Nevertheless, using a refinement of the approach first developed by Manning (8), credible estimates of the populations of precolonial African countries have been produced by Frankema and Jerven [9], based on back-projection of UN data from the 1950s back to 1850. According to them, the population of what is now Zimbabwe was approximately 665,000 in 1850, with an annual population growth rate of just over 10 per mille. Projection even further back in time requires an assumption of earlier population growth rates. Demographic transition theory [29] holds that populations would have been almost in equilibrium (that is, with very low growth rates). Estimates of the population at earlier times can be derived by assuming plausible trajectories of growth rates in earlier periods, and suggest a probable population of between 230,000 and 275,000 in 1600. This finding is significant: estimates of the population of Zimbabwe by district in 1921 [9, 27] suggest that Victoria District (in which Great Zimbabwe is situated) accounted for no more than about 5 percent of the national population. While the actual numbers may have been undercounted, provided the undercount at national level and in Victoria were roughly equivalent, the proportion offers a reasonable measure of the population distribution. Beach further argues that there is little evidence that the distribution of the population is unlikely to have changed in precolonial times. On this basis, and applying that proportion to the estimate of the population in 1600 suggests that the entire Victoria district might have had a population of between 11,500 and 14,000. On this basis alone, the suggestion that the population of Great Zimbabwe (even at its height, covering a matter of a few square kilometres in a district of 56,667 square kilometres) was greater than this, is untenable.

Second, we consider the ecological and sustainability implications of various population estimates on key resources such as land for agriculture and animal husbandry, starting from the estimate of 20,000 people. Two hundred and twenty-two rural households from three communal areas in northern and southern Zimbabwe owned on average 6.91 hectares of arable land each in the year 2005, converting into a total of 1,534 hectares, excluding pastures [25]. We are alert to the fact that colonialism changed land tenure practices, but the figures are sufficient for comparative purposes. The 20,000 people at Great Zimbabwe translates to 2,850 households based on seven per household and 20,000 ha (200 km^2^), a circle of eight square kilometres. If we create circles around Great Zimbabwe based on the methodology of Vita-Finzi and Higgs [30], a 5-, 10- and 15 km radius from the centre translates respectively into 78.54 square kilometres, 314 square kilometers, and 706.85 square kilometres of land. However, not all land around Great Zimbabwe is arable although some areas would have been cultivatable using traditional methods [31]. By comparison, low populations predicted in Model 2 suggests that the land would have been more than sufficient at 5-, 10- and 15 km distances although communal farmers rarely travel more than five kilometres in search of land for cultivation [24].

Third, we can ask how much land would have been required to sustain cattle for a population of 20,000. On average, modern households in communal areas of Zimbabwe own four cattle [32] which implies between 11,500 and 20,000 cattle for the assumed number of homesteads at Great Zimbabwe. Rattray established that grasses around Great Zimbabwe fall into sweet- and sourveld [33]. Sourveld describes grazing where animals gain weight during the growing season but lose weight during the dry season because of the poor quality of herbage; while sweetveld refers to grazing where animals gain weight during the growing season and in winter, and are able to at least maintain their body weight. Both sweet- and sourveld are found in the areas surrounding Great Zimbabwe. It is possible that the inhabitants of Great Zimbabwe practised livestock transhumance to take advantage of both types of vegetation [34]. However, since the stocking rate in the area around Great Zimbabwe is 1 Livestock Unit, per four or five hectares of pasture, the suggestion is that large herds of cattle would have been difficult to sustain [33, 34]. Any intensive cattle husbandry would have had devastating consequences for the environment. Even if cattle were loaned for diplomatic alliances [30] and large herds were not always present at the capital, the amount of land required seems implausible.

Fourth, as agricultural and metal using communities, iron would have played a significant role at Great Zimbabwe. The iron requirements of a population of 20,000 people, the majority of whom were adults, would have been enormous. If the production took place around the site, this would have created huge dumps of production debris similar to those of places such as Meroe [35, 36, 37]. In fact, the small-scale metal production remains found in and around Great Zimbabwe points more towards dispersed homestead based production. It is also possible that Great Zimbabwe might have obtained its iron, gold and copper through normal trade and exchange and tribute [35] with surplus obtained in this way channelled to the Indian Ocean trade network.

Finally, the expectation is that large populations of settled communities would leave remains of an equivalent scale while low populations will also leave remains that are commensurate with that scale. The only archaeologically outstanding remains are the stone walls, that housed a few people. Excavations of all periods have produced very few artefacts per cubic metre of deposit inside and outside the walls which is consistent with low populations. The huts at Great Zimbabwe were all single-storey structures, built of materials that left permanent marks. Even if we stretch our imaginations wildly to argue that they might have been temporary houses made of perishable material, these too would have left floors and hearths which are archaeologically recoverable. The estimate that Great Zimbabwe had a population of 20,000 people is not supported by the sparse archaeological evidence. In fact, a high population for Great Zimbabwe would make it an anomaly, a densely populated island on a sparsely occupied and predominantly rural landscape [8, 9].

## Discussion and conclusion

While all pointers are towards the observation that Great Zimbabwe’s population was very low, the dominant explanatory models were initially framed within the fictional idea of Prester John and Rider Haggard’s ‘lost cities’, and later within that of great empires such as Rome and China. Besides being densely populated, capitals such as Rome had clearly defined craft specialisation and division of labour. Historically, there is no evidence of very big towns and empires in southern Zambezia [38]. In the Shona language, towns are referred to as *maguta* (*guta*: singular) but in most cases these were a collection of homesteads [39]. Royal palaces known as *mizinda* or *madzimbahwe* often qualified to be *maguta* because they formed a constellation of numerous homesteads. However, there was no formalised bureaucracy nor a clearly defined division of labour with individuals assuming different situational roles. Advisors to the leaders and other important officials often lived in their homesteads away from centres of power. Political succession generally followed rotation of elders or houses in the lineage, who ruled from their existing homesteads and not those of their predecessors [40]. Although there was no formal bureaucracy, the centres of power were networked with other homesteads locally and regionally. This ‘dispersed urbanism’ was based on the fact that advisors and other specialists, and common people, may as circumstances demanded, have gathered at the capitals but then returned to their residences—a practice that may explain why there are no big middens at places such as Great Zimbabwe. There was no centralised control of trade, but political leaders often levied taxes in the form of tribute [41]. Surplus siphoned in this way found its way into the Indian Ocean trading system. Thus Great Zimbabwe did not need large populations to trade locally and regionally and externally with the Indian Ocean.

Low populations are essential to achieve ecological sustainability. If the population of Great Zimbabwe was high, we ought to be seeing the associated environmental consequences such as intensified erosion within the site’s resource catchment area. Land, water and pastures were readily available. In fact, low population pressure meant that forests for wood, fields and pastures, could be rotated giving them a chance to recover. Low populations would explain why no evidence of massive environmental degradation has been found within or outside Great Zimbabwe. In terms of hygiene and sanitation, it is impossible to have 20,000 people living in one place for over a hundred years without huge impacts in terms of disease. Thus, while the population of Great Zimbabwe was comparatively higher by local standards, it would still have been low enough for the economics and general health to work.

No evidence of human remains or burials have ever been found at Great Zimbabwe, yet indications from historical censuses, as well as demographic reconstruction, suggest that infant and child mortality would have been very high, perhaps of the order of 300 to 400 deaths before the age of five years from every 1,000 births. This observation is given more weight by the recovery of 96 burials from the archaeological site of K2 (CE1000–1220), located in the Shashe-Limpopo area where the three countries of Botswana, South Africa and Zimbabwe meet. Of these burials, 54 were infants whose age ranged from zero to three years [42]. The evidence from other sites in the region also points to high infant mortality. Populations in southern Zambezia only started to increase from the 1950s when advances in medical care began to be felt in the colonies. Therefore, the sustainability model in operation in precolonial southern Zambezia was based on small populations that occupied large areas to allow for shifting or alternating cultivation, livestock transhumance, and other activities that ensured that the land recovered quickly enough to be sustainable in the medium- to long term.

Our estimates and consistency checks indicate that Great Zimbabwe lacked infrastructure for sustaining high populations. Whatever the population was, it was clearly not comparable with that of modern Hong Kong as the generally accepted 20,000 would imply. That southern Zambezia historically had low populations, which only increased from 1950 onwards [43] would suggest that Great Zimbabwe, like surrounding areas, also had low populations. Therefore, it is unlikely that the abandonment of Great Zimbabwe was an outcome of the negative ecological consequences stemming from high populations. Archaeological evidence suggests that Great Zimbabwe was never abandoned, such that its longevity and resilience was likely based on maintaining a good ecological balance between low population and available resources: water, land, and pastures. Mineral wealth would have been obtained through redistribution mechanisms such as tribute, and normal trade and exchange relationships.

Finally, a low population estimate does not diminish the significance of Great Zimbabwe—rather, it makes it even greater. The scale of the achievement in drystone masonry when viewed against a background of low populations is outstanding; which poses essential questions about the amount of labour and time required to build the site. Of course, we have to understand that like Rome, Great Zimbabwe was not built in a day, but over many centuries. If we compare our method to that of Huffman and Garlake, we note that without inflating the implied figures from hut counts by a factor from an inappropriate modern population pyramid, the first part of Huffman’s method produces a low population of betweem 4 and 5 000, double that suggested by Garlake and which is closer to our Model 1. The multiple comparators used in our study achieve resonance with Garlake’s view that Great Zimbabwe was occupied by what we may today describe as low populations. However, these low populations were certainly larger than those of other contemporary settlements, which underscores the fact that Great Zimbabwe was urban. Whichever way we look at it, 18–20,000 people would have been difficult to sustain at Great Zimbabwe given the nature of available resources and a lack of supporting infrastructure, in addition to other archaeological indicators at the site.
